# ADHD as a circadian rhythm disorder: evidence and implications for chronotherapy

**DOI:** 10.3389/fpsyt.2025.1697900

**Published:** 2025-12-10

**Authors:** Brandon Luu, Nicholas Fabiano

**Affiliations:** 1 Department of Respirology, Queens University, Kingston, ON, Canada; 2 University of Ottawa, Department of Psychiatry, Ottawa, ON, Canada

**Keywords:** attention deficit and hyperactivity disorder (ADHD), circadian rhythm disorder, chronotype (morningness-eveningness), chronotherapy, insomnia

## Abstract

Accumulating evidence indicates that circadian rhythm dysfunction is a clinically significant and highly prevalent phenotype in a substantial subgroup of individuals with Attention-Deficit/Hyperactivity Disorder (ADHD). This perspective synthesizes convergent lines of evidence demonstrating strong associations between ADHD and evening chronotype with phase-delayed biological markers. Sleep disturbances are profound: insomnia and sleep disturbances affect up to 80% of adults with ADHD and similarly up to 82% of children with ADHD, delayed sleep-wake timing occurs in up to 78%, and dim-light melatonin onset (DLMO) is delayed by approximately 45 minutes in children and 90 minutes in adults. These alterations coincide with blunted and delayed cortisol rhythms, reduced pineal volume, and attenuated peripheral clock-gene rhythms (BMAL1/PER2). Intervention studies demonstrate that the circadian phase can be successfully advanced in ADHD populations. Melatonin and bright light therapy has advanced DLMO in both children and adults with ADHD. Emerging data correlate phase advancement with ADHD symptom improvement, and winter trials suggest circadian preference shifts best predict symptom improvement. Sleep programs improve ADHD symptoms, sleep quality, and functioning in children. Exercise and multimodal protocols for evening chronotypes successfully advance circadian timing in non-ADHD populations and warrant investigation in ADHD. Based on this evidence, we propose a pragmatic, behavioral-first clinical pathway: routine screening for sleep/circadian disturbances; phenotypic characterization through chronotype assessment, sleep tracking, and DLMO when feasible; implementation of fixed wake times, morning bright light exposure, evening light restriction with screen hygiene, and regularized zeitgebers; and selective low-dose melatonin for confirmed or probable DLMO delays.

## Introduction

Attention-Deficit/Hyperactivity Disorder (ADHD) is a common neurodevelopmental disorder characterized by impaired levels of inattention, hyperactivity, and impulsivity (1). There is a growing body of research identifying that ADHD has a significant sleep and circadian component (2, 3). Insomnia is present in up to 80% of adults with ADHD and similarly high rates (up to 82%) of children with ADHD (4, 5). Converging evidence indicates that circadian rhythm disruption represents a highly prevalent and clinically important phenotype that interacts with ADHD symptoms in complex, bidirectional ways in a substantial proportion (though not all) of individuals. In parallel, clinical trials have begun targeting the circadian system and have demonstrated that phase shifting the internal clock of people with ADHD can improve symptoms.

Herein, we propose the adoption of behavioral circadian interventions as adjuncts in ADHD care. This circadian-informed approach is a pragmatic, scalable, and generally low risk. We invite rigorously future well-designed, stratified trials to quantify effects on core ADHD outcomes, define responder phenotypes, and optimize circadian-focused protocols.

## Sleep disturbances, evening chronotype and delayed circadian phase in ADHD

An estimated 73-78% of children and adults with ADHD have a delayed sleep/wake cycle (6). These findings persist even in the absence of comorbid mental health conditions, with multiple independent studies confirming elevated rates of self-reported sleep problems in adults with ADHD (7). These subjective reports are corroborated by objective sleep studies, which demonstrate that sleep onset latency and sleep efficiency problems remain significantly associated with ADHD even after controlling for anxiety and depression (8).

A comprehensive systematic review found robust evidence for evening chronotype predominance in ADHD (2). Approximately three-quarters of adults who developed ADHD in childhood show objective evidence of phase-delayed circadian rhythms. Biological markers such as dim-light melatonin onset in saliva, core body temperature rhythms, and actigraphically-recorded sleep patterns are typically shifted later by roughly 90 minutes compared to neurotypical adults (9).

## Biological circadian markers and mechanisms in ADHD

### Melatonin

**Figure 1:**
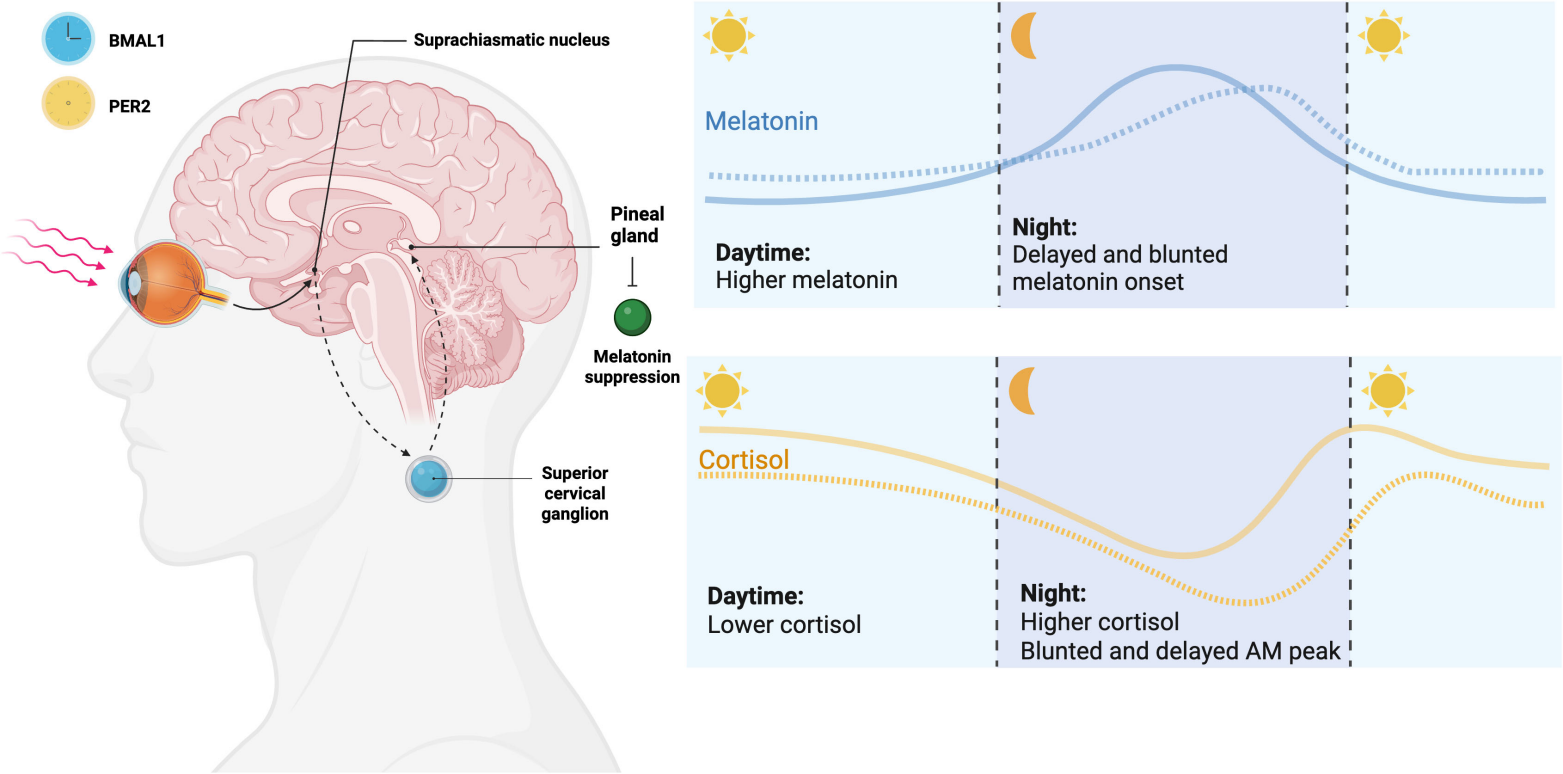
Circadian hormone profiles (melatonin and cortisol) in ADHD. Conceptual 24-h curves (dashed = ADHD; solid = neurotypical controls) illustrate a later dim-light melatonin onset (DLMO), reduced nocturnal melatonin amplitude, and occasionally higher daytime melatonin levels, alongside a later and flatter morning cortisol rise/peak in ADHD. Curves are conceptual (not to scale), and individual findings vary by age, comorbidity, and light exposure. This figure was created with BioRender.com.

Phase delays of melatonin in both individuals with ADHD have been well characterized, with children and adults identified to have a delayed onset of about 45 minutes and 90 minutes, respectively (10). Beyond the phase delay of melatonin onset in ADHD, there is evidence that the amount and pattern of melatonin production may differ (**Figure 1**). Some studies have observed abnormally high levels of melatonin during the day in children with ADHD, which improves with methylphenidate treatment (6). This ability to suppress daytime melatonin levels and shift the melatonin rhythm earlier suggests a complex interplay between ADHD medications and circadian systems. Individuals with ADHD have also been found to have smaller pineal gland (which produces and secretes melatonin) volume compared to healthy controls, with a positive correlation between pineal gland volume and eveningness (11).

### Cortisol

ADHD also involves blunted and delayed cortisol rhythms (**Figure 1**) (12, 13). In an analysis of adults with ADHD compared to age- and sex-matched controls, adults with ADHD had significantly disturbed rhythmicity of not only melatonin but cortisol (12). A meta-analysis also identified that children with ADHD exhibit lower basal cortisol levels, particularly in the morning, compared to controls (13). This suggests a deficit in the suprachiasmatic nucleus’ ability to entrain a normal circadian rhythm with alterations in the circadian rhythm expanding past melatonin. However, the causation of this is still unclear, as it has been postulated that more eveningness and thus light exposure could also be driving these findings.

### Clock gene expression

Brain and Muscle ARNT-like 1 (BMAL1) and Period circadian protein homolog 2 (PER2) are core components of the circadian clock in humans, which form a feedback loop to control gene expression in a cyclical manner. Downstream, attenuated BMAL1/PER2 rhythms in oral mucosa indicate weaker or desynchronized peripheral clocks, and symptom severity tracks with reduced PER2 rhythmicity, tying molecular clock strength to the clinical phenotype (12). Together, these data support a model in which individuals with ADHD consistently experience shifted endocrine signals (melatonin/cortisol) coupled with molecular disruptions via the loss of the rhythmic expression of clock genes.

## Clinical interventions: chronotherapy in ADHD

### Melatonin supplementation

In a randomized trial of adults with ADHD, 0.5 mg per night of melatonin advanced DLMO by 88 minutes with 14% reduction in ADHD symptoms (14). In a randomized, placebo-controlled trial of 101 medication-free children with ADHD and chronic sleep-onset insomnia, 3–6 mg melatonin nightly for 4 weeks advanced DLMO by 44 minutes, while the control group had a delay of 13 minutes. Total sleep time also significantly improved by 20 minutes with melatonin, versus a loss of 14 minutes in the control group. However, within this 4 week period, there was no identified changes in cognitive performance or problem behavior (15). In a long-term follow up study of this cohort, 65% of participants continued daily melatonin; discontinuation resulted in a phase delay of sleep in 92% of children (16). Positive improvements in behavior (71%) and mood (61%) were reported. Altogether, these findings suggest melatonin effectively advances circadian phase in ADHD; however, further trials are needed to define optimal dose and timing relative to DLMO, the duration required for sleep/phase gains to translate into core symptom improvement, and responder phenotypes.

### Bright light therapy

Studies in healthy populations have demonstrated a powerful ability to phase advance DLMO using bright light exposure. A week of natural light-dark cycle was able to entrain a ~2.6h earlier DLMO in healthy adults (17). Emerging research indicates that morning bright light can help stabilize sleep and circadian rhythms in ADHD (14, 18, 19). Adding bright light therapy to melatonin yielded the largest phase advance (~2 hours) in adults with ADHD and delayed sleep phase (14). In another pilot trial, 2 weeks of morning bright light therapy with a 10,000 lux lamp phase advanced DLMO by 31 minutes and mid-sleep time by 57 minutes in adults with ADHD. There was a significant interaction between ADHD-Rating Scales and Hyperactive-Impulsive sub-scores with phase advances in DLMO and mid-sleep time (18).

A 3-week intervention examined the role of bright light therapy in adults with ADHD during the fall/winter period (19). Phase advance in circadian preference emerged as the strongest independent predictor of improvement across subjective and objective ADHD indices. These findings, especially in the winter period, are especially relevant as there is a strong relationship between ADHD symptoms and symptoms of seasonal depression (20). One analysis suggested that circadian disturbance significantly mediated the relationship between ADHD and seasonal symptoms of depression (20). In clinical populations, the overall rate of seasonal affective disorder is 27% among adults with ADHD, with females at the highest risk (21). While more trials are required, bright light therapy may be even more efficacious for individuals with ADHD during winter months (20).

### Behavioral interventions

In a randomized trial of 244 children with ADHD, a behavioral sleep intervention (two fortnightly sessions with strategies provided by psychologists or pediatricians and a follow-up telephone call) significantly improved severity of ADHD symptoms, sleep, quality of life, behavior, and functioning at 6 months post intervention (22).

Although not explicitly examined in individuals with ADHD, exercise is another adjunctive tool to help improve circadian misalignment. Both morning or evening exercise seem to advance DLMO in those with later chronotypes, suggesting exercise at any time of day could be a useful adjunct for those with ADHD who tend to have later chronotypes (23, 24).

A multimodal approach to behavioral therapy has demonstrated significant benefits in a group of “night owls”, which may also translate to those with ADHD. Researchers randomized a group of 22 healthy individuals with late chronotypes to behavioral interventions aimed at advancing their circadian rhythm (25). The general principles were to wake up 2–3 hours earlier, wake up at the same time every day, maximize morning light exposure, reduce evening light exposure, avoid late dinners, avoid caffeine after 15:00, exercise in the morning, and avoid naps in the late afternoon. In just 3 weeks, the intervention group shifted their DLMO by ~2 hours, wake-up time advanced 1.9 hours, and peak cortisol advanced by 2.2 hours. Additionally, subjective depression scores decreased by ~58% and stress scores by ~40% in the intervention group. Cognitive performance and physical performance also improved. Overall, this could be an effective, low-cost strategy to provide structured guidance to those with ADHD, though specific trials in this population are needed.

## Conclusion

The accumulated evidence demonstrates that circadian rhythm dysfunction is highly prevalent and clinically meaningful in a substantial proportion of individuals with ADHD, although not universal, and its interaction with ADHD symptoms appears complex and bidirectional. The prevalence of circadian alterations (affecting 73-80% of ADHD patients), consistency of biological markers across studies (phase delays, altered melatonin and cortisol rhythms, disrupted clock gene expression), and efficacy of circadian-targeted interventions in improving both sleep and core ADHD symptoms support a model wherein circadian disruption may play an important role in ADHD pathophysiology in a substantial subgroup, though evidence on remission of ADHD with circadian interventions is lacking.

This evidence warrants reconsideration of current assessment and treatment paradigms. Implementation of routine circadian phenotyping in ADHD evaluation, coupled with evidence-based chronotherapeutic interventions, represents a pragmatic approach to improving outcomes. While not proposing that ADHD be reclassified exclusively as a circadian disorder, the evidence supports recognition of a prevalent circadian phenotype that, when present, may benefit from targeted chronotherapeutic intervention alongside standard ADHD treatments.

The safety profile, accessibility, and potential for synergy with existing treatments make circadian interventions an attractive addition to the ADHD treatment. As the field advances toward precision medicine approaches, circadian phenotyping may prove essential for treatment selection and optimization. Further research is needed to fully elucidate the bidirectional relationships between circadian disruption and ADHD symptoms, identify biomarkers for treatment selection, and establish optimal long-term management strategies.

## Data Availability

The original contributions presented in the study are included in the article/supplementary material. Further inquiries can be directed to the corresponding author.
